# Quantifying natural amyloid plaque accumulation using disease progression modeling with ADNI, BioFINDER, and LEARN datasets

**DOI:** 10.1002/alz70856_103495

**Published:** 2025-12-25

**Authors:** Kay Hoong Chow, Tongrong Wang, Ilke Tunali, Samantha C. Burnham, Sergey Shcherbinin, Kevin Biglan, Yvonne Vandenburg, Matan Dabora, Ivelina Gueorguieva

**Affiliations:** ^1^ Eli Lilly and Company, Bracknell, Berkshire, United Kingdom; ^2^ Eli Lilly, Indianapolis, IN, USA; ^3^ Eli Lilly and Company, Indianapolis, IN, USA

## Abstract

**Background:**

Preventing amyloid accumulation may delay the earliest stages of AD (preclinical, stages 1‐2) (Jack CR Jr et al., *Alzh Dementia* 20: 5143‐5169, 2024) and downstream irreversible pathological changes, that ultimately lead to later AD clinical stages (3+) with cognitive and functional impairment. Understanding the natural rate of amyloid plaque accumulation and timely treatment with amyloid targeting agents will be of benefit for AD prevention. Some studies using ADNI (e.g. Elhefnawy et al. *J Pharmacokin. Pharmacodyn*, Jan 2024 and Jagust WJ et al., *Neurology* 96(9):e1347, 2021) have estimated baseline and intrinsic rate of accumulation of amyloid plaque over time with one study of 10 year data suggesting it follows an exponential growth model with a population mean intrinsic rate of 3 centiloids/year, where baseline was impacted by age, gender, *APOE*‐ε4 genotype, and disease stage. The aim of this analysis is to establish plaque accumulation model over 10+ years using natural history cohorts across ADNI, BioFINDER and LEARN using novel disease progression methods and identify significant factors across a large number of participants.

**Method:**

Natural plaque accumulation model was established across the continuum of AD. Non‐linear mixed effects analyses with covariate identification used stepwise covariate selection (scm) and artificial intelligence (AI) methods and included participants from ADNI (*N* = 1745), BioFINDER (*N* = 265) and LEARN (*N* = 4492). Linear, exponential and non‐linear models were evaluated to estimate population mean and between‐participant variability on the accumulation rate, baseline, and other model parameters.

**Result:**

Age, baseline amyloid plaque, and APOE4 genotype were identified as important factors for amyloid plaque accumulation. Around the age of 60 years there was a steep transition in amyloid plaque (centiloids) followed by a slow saturation at higher ages with these changes starting at slightly earlier ages for APOE4 carriers.

**Conclusion:**

This natural amyloid plaque model predicting the time course over decades may be used in designing and interpreting novel AD trials. Preventing amyloid accumulation would avoid the onset of preclinical AD, downstream irreversible pathological changes, and ultimately cognitive and functional impairment due to AD.

